# The Intelligent Care of Dumb Brutes

**Published:** 1885-11

**Authors:** 


### CULLINGS FROM CONTEMPORARIES.

### THE INTELLIGENT CARE OF THE DUMB BRUTES.

WE have always thought it was a shame that so little attention—or rather intelligent attention—is given to the treatment of our domestic animals in sickness—seeing they are so necessary to our comfort, happiness and well-being; and that it is absurd that the opinion should prevail, as it evidently does, that to study the diseases of the lower animals is derogatory to the character and dignity of a physician. The horse and dog especially are man's most intimate companions, and at the same time servants; and the amount of intelligence displayed by them strikes us sometimes with surprise; and evidences of their friendship—their devotion to their owners is most touching. The owner of a good horse or dog is his god. An intelligent dog looks up to his master for friendship, care and protection, and evidently thinks that there is nothing on earth higher, better, nobler than he. How do we repay this devotion, and the service of our dumb friend in time of sickness? We know nothing of his anatomy, his physiology, or pathology, nor of the diseases to which dog flesh and horse flesh are heir; and there is no one who does. You cannot send your family physivian to prescribe for him, even though he be a $50,000 Dexter, for, in the first place, said f. p. would get on his dignity and feel affronted; and, in the second place, he knows nothing about dog diseases or horse diseases if he were to go. The livery stable horse doctor is the only available skill in most localities; in some cities—very few—there are intelligent veterinarians, but not *thoroughly* educated veterinarians. Such are rarer in the South than the proverbial hen's teeth. We know from experience with sick horses and sick dogs of our own, about how much skill is possessed by the average veterinarian in this country. I legal friend of ours, who owned a beautiful imported pointer, asked us to see and prescribe for his dog, on one occasion; and we, holding the views just expressed, and appreciating his sympathy and fondness for his dog, did not feel insulted. Our friend made a remark at the time which struck us with great force. He said: "Doctor, the more I see of men, the better I like dogs."

We are much gratified to see that the want for educated veterinarians is recognized, and is to be supplied, in part at least, and we reproduce the following from C. W. D.'s letter in the *Journal of the American Medical Association* with much satisfaction:

"The Veterinary Department of the University of Pennsylvania is now fully opened. It has been organized at an expense of about fifty thousand dollars, with Dr. Huidekoper, who studied veterinary medicine in Europe, after having graduated in medicine in the University some years before, at its head. The buildings now completed are extensive and well suited to their object. They are of stone and brick, and extend for more that four hundred feet along Pine street, which passes through the grounds of the University. They contain stalls, seat-baths, foot-baths, a paded cell for lunatics horses, an armory for instruments, forges, lecture rooms, working labratories, dissecting room, and in fact, everything necessary for studying the diseases of the lower animals under the most favorable circumstances. A large part of the building is to be used as a hospital for sick and injured horses, cattle, sheep, dogs, cats, etc.

It is to be hoped that the attention to be given to the study of veterinary medicine, in this department of the University, will lead to an advance in the position which this art shall occupy in this country. In Europe there are a number of men of high scientific attainments who are veterinarians, and their contributions to the art of healing have a much wider range than that of the class which is especially the object of their study. There is no reason why the study of comparative medicine should not receive a new impetus from the association of this new department with the others already existing in the University. It is to be hoped also, that the study of veterinary medicine under these auspices, will attract a class of men who are capable of elevating the pursuit to which they are to devote themselves above the level which it has hitherto occupied in this country. It is a calling which stands in need of men who will consider it honorable and help to make it so. To young men with zeal and perseverence it offers a most promising field of scientific study, as well as an unusual certain road toward a good income. It would probably be good for both man and beast, if some of the young men who are now thinking about studying medicine would make up their minds to study animal medicine, in which they would have a clearer field to themselves, and leave a little clearer field to those who are now trying to make a living out of their fellow men."

